# Incremental levels of diagnostic information incentivize health-seeking in non-alcoholic fatty liver: a randomized clinical trial

**DOI:** 10.1038/s41598-022-12295-1

**Published:** 2022-05-18

**Authors:** Norberto C. Chavez-Tapia, Tonatiuh Barrientos-Gutierrez, Leticia Torres-Ibarra, Beatriz Sanchez-Jiménez, Eva Juarez-Hernandez, Martha Ramos-Ostos, Luis F. Alva-Lopez, Misael Uribe

**Affiliations:** 1 Obesity and Digestive Diseases Unit and Translational Research Unit, Medica Sur Clinic and Foundation, Puente de Piedra 150 Col. Toriello Guerra Tlalpan, 14050 Mexico City, Mexico; 2 Center for Population Health Research, National Institute of Public Health, Cuernavaca, Mexico; 3 Gastroenterology Department, Centro Medico Nacional La Raza, Instituto Mexicano del Seguro Social, Mexico City, Mexico; 4 Translational Research Unit, Medica Sur Clinic and Foundation, Mexico City, Mexico; 5 Integral Diagnosis and Treatment Center, Medica Sur Clinic and Foundation, Mexico City, Mexico; 6 Radiology and Medical Imaging Unit, Medica Sur Clinic and Foundation, Mexico City, Mexico

**Keywords:** Medical research, Gastrointestinal diseases, Hepatology

## Abstract

Patients with chronic disorders like non-alcoholic fatty liver disease (NAFLD) face important challenges adhering to diagnostic and treatment tracks. As NAFLD increases, the need to incentivize health-seeking behaviors grows. No evidence-based interventions to address this gap exist. The aim of the study was to estimate the effect of providing increasing levels of diagnostic information on medical care-seeking in adults newly diagnosed with NAFLD. We randomly assigned adults with a sonographic diagnosis of NAFLD at a check-up unit in Mexico to one of five groups. All groups received medical consultation. A: no further interventions; B: received multimedia educational material (MEM); C: MEM + NAFLD-fibrosis-score (NFS); D: MEM + transient elastography (TE); E: MEM + NFS + TE. 1209 participants were randomized, follow-up rate 91%; 82% male, BMI 30.5 ± 4 kg/m^2^. There were no differences in the proportion of patients undergoing further diagnostic evaluation of liver fibrosis (A 0.4%, E 0.4%, P-for-trend = 0.269). Groups who received more information sought specialized medical care more frequently: A 22%, E 30% (P-for-trend = 0.047). A trend to receive treatment was also observed at higher levels of information: A 26.7%, E 36.3% (P-for-trend = 0.134). Increasing the amount of diagnostic information seemed to increase patient’s health-seeking. Tailoring the communication of information obtained for diagnosis could help to increase health-seeking in chronic disease patients.

Trial registration: NCT01874249 (full date of first registration 11-06-2013).

## Introduction

Non-alcoholic fatty liver disease (NAFLD) is highly prevalent. Complex mechanisms to explain the pathophysiology of NAFLD have been proposed, yet, overweight and insulin resistance remain at the core. Consequently, the therapeutic approach is mainly based on exercise and diet, with limited impact due to lack of patient compliance^1^. According to the latest meta-analysis, only 4.4–8.8% of patients assigned to diet modification and exercise reduced their body weight^2^, thus, a limited impact on liver-related outcomes has been noted^3^. Effective interventions to prevent the course of NAFLD are urgently needed, considering that NAFLD is expected to become the most common cause of liver fibrosis, cirrhosis, and transplantation^4^.

Liver fibrosis is a complication of NAFLD and an early indicator of progression towards cirrhosis. Current NAFLD guidelines encourage the use of non-invasive methods over more invasive and harmful approaches (such as liver biopsy) to assess liver fibrosis^5^. Even though the severity of fibrosis is directly related to mortality, few interventions had proved to limit fibrosis progression; therefore, the diagnosis of liver fibrosis has been criticized as providing little added value, while significantly increasing the healthcare cost.

Despite that lifestyle changes remain the therapeutic target, regardless of the fibrosis stage, the influence of diagnostic information produced with different degrees of technology in seeking medical care has been scarcely studied^6,7^. A potential effect of liver fibrosis assessment that has not been quantified is how having more information about additional medical tests for liver fibrosis could influence their awareness and management of NAFLD promoting health-seeking behaviors. In coronary artery disease, knowledge about coronary artery calcium has been associated with health-promoting behaviors and increased adherence to pharmacological treatment, suggesting that providing timely diagnostic information to patients could help to achieve therapeutic targets^8^.

Adherence to recommendations for lifestyle modifications in NAFLD is a frequent problem, as it is with any chronic disease, and awareness about the disease and willingness to comply with the treatment is paramount to therapeutic success. Still, it is unknown if increasing the amount of knowledge about liver fibrosis could improve health-seeking behaviors in NAFLD patients.

This randomized clinical trial aimed to evaluate the effect of communicating increasing levels of information about liver fibrosis on health-seeking behaviors using non-invasive tests. We hypothesized that patients receiving increasing and more detailed information about liver fibrosis would seek more medical care for the management and treatment of their disease after 1 year follow-up vs. comparison group participants receiving less level of information.

## Methods

We randomly assigned asymptomatic adults with NAFLD to one of five interventions with increasing levels of diagnostic information. One year after diagnosis we assessed if participants sought further diagnosis of liver fibrosis, if they received a specialist consultation, or if they received any kind of treatment for NAFLD.

### Trial design

This is an open-label randomized clinical trial of five parallel groups with a 1:1 allocation ratio (Clinical trials registration NCT01874249).

### Eligibility criteria for participants

Every adult (18–65 years old), who participated in a voluntary general preventive medical evaluation at a hospital in Mexico City (Medica Sur Clinic and Foundation) from June 2013 to June 2016, with overweight (BMI > 27 kg/m^2^), and diagnosis of NAFLD by ultrasound. Exclusion criteria comprised: subjects who reported a previous diagnosis of liver disease; alcohol consumption higher than 140 g/week in men, and 70 g/week in women; risk factors for viral hepatitis; past or current use of tamoxifen, methotrexate, amiodarone, diltiazem, or any antiretroviral therapy. Eligible patients were invited to participate in this trial just after the ultrasonographic diagnosis of liver steatosis was done.

### Setting

Patients went to a voluntarily medical assessment in a private hospital. As this is a private institution, in case a patient has NAFLD all patients receive concealing about management, but the final decision is based on patient preferences.

### Interventions

The overall goal of the four intervention groups was to promote medical care-seeking among newly diagnosed NAFLD patients by increasing the level of diagnosis information provided during routine medical care visits. The content for each one of the four interventions was based on the transtheoretical model of behavior change^9^. Multimedia educational material was provided in a compact disc and contained a PowerPoint presentation with 32 slides with images and information about NAFLD, its risk factors, diagnosis, preventive strategies, management, treatment, complications, and prognosis. The NAFLD fibrosis score was calculated as: − 1.675 + 0.037 × age (years) + 0.094 × BMI (kg/m^2^) + 1.13 × IFG/diabetes (yes = 1, no = 0) + 0.99 × AST/ALT ratio − 0.013 × platelet (× 10^9^/l) − 0.66 × albumin (g/dL). The cut-off for advanced fibrosis was a score > 0.675^10^. Transient elastography was performed with M or XL probes; to ensure the reliability of the study we considered: (1) at least ten valid measurements, (2) ≥ 60% success rate, and (3) interquartile range/median ratio (IQR/M) ≤ 30%. The cut-off to select a probe was ≥ 35 mm from skin to liver measured with abdominal sonography for the XL probe and < 35 mm for the M probe. Fibrosis score was based on the criteria proposed by Wong, being advanced fibrosis with a cut-off > 8.7 kPa^11^.

Al patients receive a structured medical consultation in which all diagnoses detected during the check-up (including NAFLD) were discussed for at least 30 min. Written informed consent was obtained before enrollment, and the Research Ethics Board of Medical Sur Clinic and Foundation approved the protocol, all methods were carried out in accordance with relevant guidelines and regulations.

### Outcomes

We hypothesized that providing increasing amounts of information from non-invasive diagnostic procedures for fibrosis would help move patients from the contemplation and preparation stages to the action stage, according to the transtheoretical model^9^. We expected that higher levels of information would increase the interest of patients to continue on their diagnostic track, ultimately reflected by a higher rate of liver fibrosis diagnosis in the group receiving more information. Consequently, the primary outcome was the proportion of patients who self-reported undergone further invasive or non-invasive tests of liver fibrosis diagnosis 1 year after randomization (e.g. asking specifically for liver biopsy or other non-invasive methods for assessment and staging of liver fibrosis). The secondary outcome was the proportion of patients who sought specialized medical consultation (gastroenterologist or endocrinologist) or treatment for NAFLD (including nutritional assessment, physical activity, any drug prescribed for NAFLD, or surgical management of obesity). The outcomes were assessed through a telephone call, 1 year after baseline; when unavailable, an email was sent to contact the patients.

### Sample size

208 patients per group were needed to identify a 5% increase in the number of patients who self-reported undergone further invasive or non-invasive tests of liver fibrosis diagnosis 1 year after randomization, with an 80% power and 5% alpha, two-sided. No previous data exists reporting the percentage of people with a diagnosis of NAFLD that seek a further diagnosis of liver fibrosis in any period. Screening studies in NAFLD have shown a prevalence of advanced fibrosis of 5%^12^.

### Randomization

**Figure 1:**
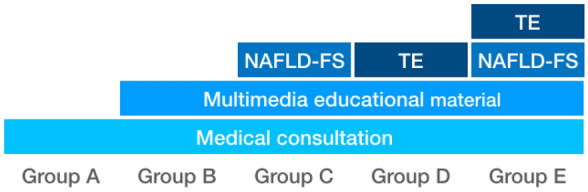
Interventions received per group. Non alcoholic fatty liver disease fibrosis score (NAFLD-FS), transient elastography (TE).

A central office generated a simple randomization sequence using a computer-based random number generator. Allocation information was stored in individual envelopes at the central office, that was transported to check-up centers. An external physician enrolled the patients and opened the envelopes at the time of intervention assignment. Patients were randomly assigned to one of five groups. All groups received a structural medical consultation in which all diagnoses detected during the check-up (including NAFLD) were discussed for at least 30 min; group B received multimedia educational material (MEM) with detailed information about NAFLD; group C received MEM plus assessment of liver fibrosis using NAFLD fibrosis score; group D received MEM plus assessment of liver fibrosis using transient elastography; group E received MEM plus an assessment of liver fibrosis using both NAFLD fibrosis score and transient elastography (Fig. 1).

### Blinding

By the nature of the intervention clinicians and patients were aware of the group of the interventionafter enrrolment. During follow-up, all researchers were blinded to the group assigned to each patient. Different researchers participated separately in the recruitment and follow-up; researchers only participated in the trial for 1-year periods. Data analysis was conducted independently of field researchers and procedures by TBG and LTI.

### Statistical analysis

Descriptive analyses for continuous data were conducted using mean and standard deviation; bivariate analyses used Student *t* test or Mann–Whitney *U* test. Categorical data were reported as frequencies and percentages and analyzed by Chi-squared or Fisher’s tests. To assess the impact of increasing levels of information on monotonic increases of health-seeking behaviors we estimated P-for-trends, fitting logistic regression models. The number needed to treat (NNT) was calculated as the inverse of the absolute event reduction. Here, treatment was defined as increasing the information about non-invasive liver fibrosis tests. All analyses were conducted in STATA 13 (College Station, TX).

All authors had access to the study data and had reviewed and approved the final manuscript.

## Results

**Figure 2:**
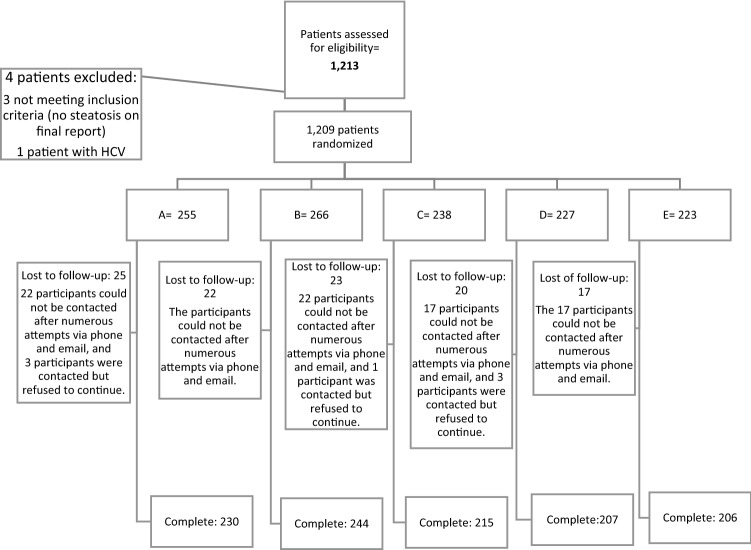
Study flow diagram.

Figure 2 presents the study flow diagram. A total of 1209 patients were randomized: 255 were assigned to group A, 266 to group B, 238 to group C, 227 to group D, and 223 to group E. Patients were recruited from June 2012 to June 2015, the follow-up period ended in June 2016, 1 year after the last patient was included.

Table 1 shows the baseline characteristics of the participants. Most patients were male (82%, n = 995), with a BMI of 30.5 ± 4 kg/m^2^, and 47% were obese (n = 563). Diabetes was found on 6% (n = 75), high blood pressure on 18% (n = 221), high blood cholesterol levels on 56% (n = 678), and metabolic syndrome on 41% (n = 491); all diagnostic criteria were based on ATP III guidelines^13^. At baseline, 1% of patients (n = 12) had advanced liver fibrosis according to NAFLD score, and 7% (n = 81) had advanced fibrosis according to transient elastography defined as ≥ 8.7 kPA. There were no differences in baseline characteristics across study arms (Table 1).

**Table 1:** Comparison between patients with and without liver fibrosis.

	Patients without fibrosis (n = 120)	Patients with fibrosis (n = 40)	*P* value
Sex (male %)	65.8	52.5	0.131
Age (mean ± SD)	54.8 ± 13.1	52.9 ± 12.9	0.418
**BMI (%)**			
Normal	19.2	7.5	0.004
Overweight	50	32.5	
Obesity	30.8	60	
Smoking (%)	27.5	17.5	0.206
Type 2 diabetes (%)	17.5	40	0.003
Hypertension (%)	27.5	35	0.367
Dyslipidemia (%)	38.3	35	0.706
Chemoprophylaxis (%)	20.5	12.8	0.286
Methotrexate (%)	67.3	56.8	0.246
Albumin mg/dL (mean ± SD)	4.4 ± 0.42	4.2 ± 0.65	0.0564
GGT UI/L (mean ± SD)	40.1 ± 39.2	70.8 ± 84.4	0.0022
Platelets × 10^9^/L (mean ± SD)	242.8 ± 49.7	210.7 ± 58.9	0.0009
Cholesterol mg/dL (mean ± SD)	195.9 ± 38.3	189.0 ± 44.2	0.3448
Tryglicerides mg/dL (mean ± SD)	179.7 ± 92.9	183.9 ± 58.7	0.7911
Years with disease (mean ± SD)	15.5 ± 9.6	17.8 ± 10.0	0.1992
NAFLD Score (mean ± SD)	0.58 ± 0.91	1.22 ± 0.92	0.0002
HSI (mean ± SD)	38.9 ± 6.4	42.2 ± 6.8	0.0054

*Chi-squares for proportion and t test for means and standard deviations. *BMI* Body Mass Index, *GGT* gamma glutamyl transpeptidase, *NAFLD* non-alcoholic fatty liver disease, *HIS* Hepatic steatosis index. Fibrosis was considered any of the following: a NAFLD fibrosis score > 0.675 and/or a transient elastography value > 8.7 kPa.

### Primary outcome: number of patients who self reported undergone further invasive or non-invasive tests for liver fibrosis diagnosis

After 1 year of follow-up, 91% (n = 1102) of participants were successfully reached to assess the effect of the intervention.

Overall, 0.2% of all patients (n = 8) sought further diagnostic assessment for liver fibrosis either with an invasive or non-invasive method (one patient in group A, two in group B, two patients in group C, three patients in group D, and one patient in group E; P-for-trend = 0.269); none were diagnosed with advanced liver fibrosis (Table 2).

**Table 2:** Main outcomes.

	A (n = 255)	B (n = 266)	C (n = 238)	D (n = 227)	E (n = 223)	P for trend
Seeking further diagnosis for liver fibrosis	0.4% (1)	0.4% (1)	0.8% (2)	1.3% (3)	0.4% (1)	0.269
**Seeking specialist**						
% (n)	22% (56)	27.4% (73)	27.3% (65)	29.5% (67)	30.0% (67) *	0.047
NNT		18.24	18.69	13.24	12.37	
(95% CI)		(− 52.50 to 7.77)	(− 44.32 to 7.72)	(− 388.94 to 6.51)	(6.27 to 479)	
Treatment % (n)	26.7% (68)	33.5% (89)	32.8% (78)	32.2% (73)	36.3% (81)*	0.134
NNT		14.7	16.38	18.21	10.36	
(95% CI)		(− 94.58 to 6.83)	(− 51.10 to 7.06)	(− 37.66 to 7.33)	(5.56 to 75.11)	

*TE* transient elastography, *NNS* NUmber needed to screen. Seeking specialist (gastroenterologist or endocrinologist). Treatment for NAFLD (including nutritional assessment, physical activity, any drug prescribed for NAFLD, or surgical management of obesity). *P-value ≤ 0.05 for group A vs*.* group E comparison.

### Secondary outcome: seeking medical care or treatment after the intervention

At 1 year of follow-up, participants receiving more information in their intervention group were significantly more likely than the comparison group to have sought specialized medical care: 22% in group A, 27.4% in group B, 27.3% in group C, 29.5% in group D, and 30% in group E (P-for-trend = 0.047). Particularly, when comparing the group with the largest amount of diagnostic test information vs. the standard of care, there was an 8% statistically significant increase in the medical care-seeking (P-value = 0.044). Regarding the number of patients receiving treatment for NAFLD, a trend towards more medical treatment was observed as more level of information of the intervention group: 26.7% in group A, 33.5% in group B, 32.8% in group C, 32.2% in group D, and 36.3% in group E, although this was not statistically significant (P-for-trend = 0.134). However, when contrasting the control group (group A) vs. group E, there was a significant increase in medical treatment (P = 0.023) (Table 2).

## Discussion

We aimed to estimate the effect of increasing medical information provided to patients with NAFLD in their healthcare-seeking behaviors by seeking further medical care and receiving medical treatment.

We found that increasing the level of diagnosis information provided during routine medical care visits, based on the use of non-invasive tests, led to an increase in healthcare seeking, as suggested by an increase in patients who sought medical help, and to a lesser degree to the number of patients receiving treatment for NAFLD.

The medical information provided to chronic disease patients could help to achieve care goals for their disease management by improving healthcare-seeking. In our study, we observed an eight percentage points increase in seeking specialized care between the group receiving standard information (22%) and the group receiving the most information (30%), and a 9.6% increase for receiving NAFLD treatment (26.7% and 36.3%, respectively). This is different from studies in chest pain in which patient-oriented information did not modify healthcare outcomes^14,15^.

Our findings have important clinical implications because, in many clinical care settings, a set of results from diagnostic tests along with other supporting information about certain health conditions are available during routine care. If the level of routine medical information enhances the diagnostic certainty among patients, might be possible that the shift of the pre-contemplation stage towards action, following the transtheoretical model, occurs more rapidly. Having more information about their health status may improve patient awareness, increase patient satisfaction, and motivate people to be more involved in their care make them more active during share-decision making with their providers^16^.

The impact of diagnosis information provision on patient’s behavior after the diagnosis of chronic diseases has been scarcely explored, most of the research to promote health behavior change has been focused on health counseling, online peer support, and lastly by using of technology to deliver health behavior interventions^17,18^.

Information provision has been long proposed as a potential intervention to increase patient engagement and secure long-term adherence to diagnostic tracks and treatment^19,20^. Patient education programs aimed at increased disease information or risk information or to improve communication strategies have been used in several chronic diseases, such as diabetes, hypertension, coronary heart disease^21^, palliative care^22^, and cancer^23^. But, to our knowledge, there are no data from intervention studies to promote help-seeking for patients with chronic diseases addressing to provide incremental medical test information using data that is regularly generated during routine patient care. Findings from observational studies have found that patients with chronic conditions have an interest in diagnosis information^24^. Hessen et al.^25^ reported that patients with a recent diagnosis of multiple sclerosis have a major interest in the results of the diagnostic procedures. Moreover, according to data from Husson et al.^26^ among lymphoma patients, receiving more medical test information was associated with better emotional and cognitive functioning. Whereas, a study with lymphoma survivors found that receiving more information on medical test was associated with more patient’s satisfaction^27^. Considering our results, we believe that taking advantage of the already available information from clinical charts and registries to increase the amount of information a patient receive could be a powerful tool to increase treatment and specialized medical care-seeking. Nevertheless, we acknowledge that the content of information and the communication strategies in clinical practice should take into account the motivational readiness for behavior change, patient needs as well as their health literacy and communication preferences among patients^22,28^. In fact, results from intervention studies in oncology have been noticed that beyond quantity is the quality of information the most relevant for adequate information provision^16^. Providing too much information related to lifestyle changes can overwhelm individuals^29^, which highlights the need for designing tailoring medical information that overcomes the determinants of target behaviors. Participants were largely workers with access to private insurance that included a yearly check-up; as a reflection of the labor market in Mexico, men were overrepresented.

To our knowledge, this is the first study to quantify the effect of increasing levels of diagnostic information to improve health-seeking behaviors in NAFLD patients. NAFLD increases the risk for other non-liver diseases, thus, increasing treatment could produce additional positive effects in related comorbidities, mainly those in which lifestyle interventions are key such as, type 2 diabetes, high blood pressure level, dyslipidemia, etc. However, some limitations should be mentioned, first, we failed to demonstrate an increase in further assessment for liver fibrosis; we plan to continue the patients follow up to assert this outcome later on, maybe the study is underpowered due to a lower fibrosis prevalence (observed 3% vs. expected 5%). The data collection for the outcomes at 12-months of follow-up relied on self-report and could be subject to a bias of memory or interpretation, although we have no reason to expect these potential biases to be differential across intervention groups. Additionally an end-of-study visit would be useful to provide objective information about patient-centered data and should be considered in future trials. We know that patient’s understanding of the information received could influence the examined outcomes, however, this work was focused only on the quantity of information, attempting to explore if the routine diagnosis information already available could add something by itself to the patient enhancement. Of course, we cannot assume that the level of information can be straightforwardly related to the level of patient’s understanding or with patient satisfaction. We defined a series of non-invasive tests for liver fibrosis to be conducted in our patients that may not necessarily be regularly conducted in all clinical settings; we do not advocate to conduct further non-invasive tests just to increase the level of information provided to patients, but rather to use the information that is routinely available to design communication strategies to reinforce the health-seeking behaviors of patients. Finally, NAFLD was diagnosed with ultrasound, an imperfect tool, but available in heterogenous clinical settings. It is important to mention that this study was not intented to explore the indications to perform additional testing (including liver biopsy), therapeutics or prognosis in NAFLD.

In conclusion, the number of patients who self-referred undergone further diagnostic and management procedures for NAFLD increases as more comprehensive diagnostic information is provided to them.
